# Disruption of phenylalanine hydroxylase reduces adult lifespan and fecundity, and impairs embryonic development in parthenogenetic pea aphids

**DOI:** 10.1038/srep34321

**Published:** 2016-10-03

**Authors:** Pierre Simonet, Karen Gaget, Nicolas Parisot, Gabrielle Duport, Marjolaine Rey, Gérard Febvay, Hubert Charles, Patrick Callaerts, Stefano Colella, Federica Calevro

**Affiliations:** 1 Univ Lyon, INSA-Lyon, INRA, BF2I, UMR0203, F-69621, Villeurbanne, France; 2 KU Leuven, University of Leuven, Department of Human Genetics, Laboratory of Behavioral and Developmental Genetics, B-3000, Leuven, Belgium; 3 VIB Center for the Biology of Disease, B-3000, Leuven, Belgium

## Abstract

Phenylalanine hydroxylase (PAH) is a key tyrosine-biosynthetic enzyme involved in neurological and melanin-associated physiological processes. Despite extensive investigations in holometabolous insects, a PAH contribution to insect embryonic development has never been demonstrated. Here, we have characterized, for the first time, the *PAH* gene in a hemimetabolous insect, the aphid *Acyrthosiphon pisum*. Phylogenetic and sequence analyses confirmed that ApPAH is closely related to metazoan PAH, exhibiting the typical ACT regulatory and catalytic domains. Temporal expression patterns suggest that *ApPAH* has an important role in aphid developmental physiology, its mRNA levels peaking at the end of embryonic development. We used parental ds*ApPAH* treatment to generate successful knockdown in aphid embryos and to study its developmental role. *ApPAH* inactivation shortens the adult aphid lifespan and considerably affects fecundity by diminishing the number of nymphs laid and impairing embryonic development, with newborn nymphs exhibiting severe morphological defects. Using single nymph HPLC analyses, we demonstrated a significant tyrosine deficiency and a consistent accumulation of the upstream tyrosine precursor, phenylalanine, in defective nymphs, thus confirming the RNAi-mediated disruption of PAH activity. This study provides first insights into the role of *PAH* in hemimetabolous insects and demonstrates that this metabolic gene is essential for insect embryonic development.

Phenylalanine hydroxylase (PAH; EC 1.14.16.1) is an important metabolic enzyme, belonging to the aromatic amino acid hydroxylase (AAAH) family^1^, that catalyzes the conversion of phenylalanine (Phe) to tyrosine (Tyr) in a tetrahydrobiopterin (BH4)-dependent reaction^2^. This mixed-function monooxygenase is highly conserved across distantly related organisms and it has been identified, and characterized, in prokaryotes^3^,^4^,^5^,^6^, plants^7^, fungi^8^, protozoans^9^,^10^ and metazoans^11^,^12^,^13^,^14^,^15^,^16^,^17^,^18^. Mammalian PAH has been studied intensively due to its involvement in phenylketonuria (PKU), an autosomal recessive inborn error of phenylalanine metabolism resulting in profound mental retardation, seizures, microcephaly and delayed development^19^,^20^. PAH constitutes the rate-limiting enzyme in phenylalanine catabolism and its impairment leads to a neurotoxic accumulation of phenylalanine, associated with a subsequent deficiency of tyrosine and of its catecholamine neurotransmitter derivatives (i.e. dopamine, norepinephrine and epinephrine)^21^,^22^,^23^,^24^.

Studies on non-mammalian eukaryotic models have extended the role of PAH to the biogenesis of melanin, a decisive component of animal pigmentation^15^,^18^,^25^. In particular, PAH involvement in melanin-associated physiological processes has been mainly explored in holometabolous insects that develop from immature stages to fully reproductive adults via intermediate metamorphic-pupal stages^26^. In these insects, where the gene functions have been investigated in *Drosophila*, silkworms and mosquitoes^17^,^18^,^27^,^28^,^29^,^30^,^31^,^32^, PAH is associated with the numerous changes in cuticular coloration and sclerotization that allow for the extensive reconstruction and remodeling of internal structures occurring during metamorphosis. Other, more general, PAH-related functions in holometaboulous insects are tanning of egg chorion and melanotic encapsulation of parasitic organisms. Intriguingly, there is a lack of studies focusing on the functions of *PAH* and, more globally, of other genes potentially involved in development in hemimetabolous insects^33^, the embryos of which hatch directly into nymphs phenotypically resembling miniature adults^26^. Moreover, although the studies on PAH functions in holometabolous insects have provided an extensive amount of data concerning its role during insect post-embryonic development and adult physiological processes, the PAH involvement in embryonic development in insects has not been investigated. A few reports have shown an increase in *PAH* expression during embryogenesis in the fruit fly *D. melanogaster*^14^ and the silkworm *B. mori*^18^, suggesting that this gene might also play an important role in insect embryonic development. Nevertheless, no functional analysis has investigated this role so far.

Recently, we have identified tyrosine biosynthesis as a key metabolic pathway for parthenogenetic development of the pea aphid *Acyrthosiphon pisum*^34^, a major globally distributed crop pest. This emerging model organism, whose genome was the first to be sequenced and annotated among the hemimetabolous insects^35^, provides unique opportunities for the study of genetic mechanisms regulating its embryonic development. Pea aphids can either reproduce sexually or parthenogenetically. In viviparous parthenogenetic reproductive mode, females contain tens of genetically identical embryos that develop sequentially within maternal ovarioles (see Supplementary Fig. S1). This reproductive mode is particularly suited to revealing gene knockdown-associated phenotypes and related gene functions that cannot be observed in the embryos of oviparous reproduction.

Interestingly, in this hemipteran species, PAH synthesizes tyrosine not only for the insect but also for its obligate bacterial endosymbiont, *Buchnera aphidicola* (Fig. 1). Indeed, the endosymbiont is capable of producing all the necessary precursors, but it is completely dependent on aphid PAH which compensates for the absence of the final *B. aphidicola* tyrosine-biosynthetic enzymes (TyrA and TyrB)^35^,^36^. In Rabatel *et al*.^34^, we have shown that the tyrosine biosynthesis pathway is clearly activated, with several genes being highly expressed, in the late phase of embryonic development (developmental stages 16–18 and 19–20, as described by Miura *et al*.^37^) and at the beginning of nymphal development, in parthenogenetic pea aphids. Remarkably, the *A. pisum PAH* putative gene (*ACYPI007803*), which belongs to the highly expressed gene group, shows significantly increased expression levels in late embryos and in the first nymphal stage. Consistent with this gene regulation, HPLC analyses have shown that tyrosine accumulates throughout embryonic development, supporting the hypothesis of a key function for the PAH enzyme, and its amino acid product, in the terminal phase of aphid embryogenesis.

In this study, we focused on characterizing the biological functions of the *ACYPI007803* gene in *A. pisum* using RNA interference (RNAi). Our results reveal that *ACYPI007803* encodes the *A. pisum* PAH homolog and that its knockdown disrupts different life history traits and also causes morphological defects in newborn nymphs. Single nymph HPLC analyses confirmed that these developmental impairments are related to a significant tyrosine deficiency and an accumulation of the upstream tyrosine precursor, phenylalanine, thus demonstrating the RNAi-mediated disruption of PAH activity. We conclude that PAH is essential for insect embryonic development and reveal, for the first time, its key role in hemimetabolous insect physiology.

## Results

### Sequence analysis of *A. pisum PAH*

The *ApPAH* sequence (*ACYPI007803*) was obtained from version 2 of the pea aphid genome assembly, available from AphidBase^38^ (https://www.aphidbase.com). This gene is composed of 8 exons containing a coding sequence (CDS) of 1, 359 bp (Fig. 2a). A unique canonical polyadenylation signal (AATAAA) was identified in the 3′ UTR region. The presence of a complete transcript containing all 8 predicted exons was confirmed with the available RNA-seq data from AphidBase. Contrary to mammalian species, the analysis of the *PAH* genomic organization revealed a high variability of the *PAH* exon-intron structure in insects, ranging from 3 exons in the southern house mosquito *Culex quinquefasciatus* to 11 in the alfalfa leafcutter bee *Megachile rotundata* (see Supplementary Table S1). The *ApPAH* transcript encodes a putative protein of 452 amino acid residues (ACYPI007803-PA) with a predicted molecular weight of 51.409 kDa and an isoelectric point of 5.6. As seen in the structure of other characterized PAH proteins, ApPAH contains a putative amino acid binding site in the conserved ACT regulatory domain (Gly43, Thr44, Leu45, Ala46, Glu63, Ser64, Arg65 and Ser66), a conserved tetrahydrobiopterin (BH_4_) cofactor binding site (Gly247, Leu249, Phe254, Ala322 and Tyr325), a metal binding site for iron atoms (His285, His290 and Glu330), and a substrate binding pocket (His138, Arg270, Tyr277, Thr278, Pro279, Gly280, His285, Trp326, Gly346, Ser349 and Ser350) (Fig. 2b). Sequence homology analysis revealed that the ApPAH protein shows the greatest sequence similarities with insect PAH proteins, sharing 97% amino acid sequence identity with the Russian wheat aphid *Diuraphis noxia*, 94% with the cotton aphid *Aphis gossypii*, 74% with the Asian citrus psyllid *Diaphorina citri*, 73% with the human body louse *Pediculus humanus corporis*, 67% with the fruit fly *Drosophila melanogaster* and the silkworm *Bombyx mori*, and 66% with the yellow fever mosquito *Aedes aegypti* (see Supplementary Table S1). Phylogenetic analysis confirmed that ApPAH does belong to the insect PAH family and it shows a one-to-one orthologous relationship amongst insect species (Fig. 3). Moreover, our results demonstrated a specific clustering of ApPAH with most of hemimetabolous insects and, more specifically, with hemipteran species.

### Analysis of *ApPAH* mRNA expression throughout the aphid life cycle

To characterize the developmental expression profile of *ApPAH*, we determined transcript abundance in whole aphids at different life stages by means of quantitative reverse transcription PCR (qRT-PCR). The results revealed that *ApPAH* is expressed throughout the aphid life cycle, with maximum expression levels at the end of embryonic development (late embryos LE: corresponding to embryonic stages 19–20, as described by Miura *et al*.^37^) and the beginning of nymphal development (N1 nymphal instars) (Fig. 4). *ApPAH* transcript levels in early embryos EE (embryonic stages ≤15) and intermediate embryos IE (embryonic stages 16–18), in N2, N3 and N4 nymphal instars, and in A8, A15 and A23 adults, were 21.1-, 3.5-, 6.2-, 8.3-, 3.3-, 3.0-, 3.0-, and 2.8-fold lower than in the LE embryonic stages, respectively (Tukey’s HSD test, P < 0.001). No significant differences in expression levels were found between the N1 nymphal instar and the LE embryo group. These results offer a complete overview of the *ApPAH* mRNA expression throughout the aphid life cycle, complement previously obtained expression data^34^ and support the hypothesis that *ApPAH* plays a key role in the developmental physiology of parthenogenetic aphids.

### Effects of *ApPAH* RNAi on aphid life history traits

RNAi-mediated knockdown of *ApPAH* was performed by the injection of double-stranded RNA (ds*ApPAH*) into N3 nymphal instars. We first analyzed the effect of different dsRNA concentrations on aphid survival (Fig. 5). The results revealed that injections of increasing dsRNA concentrations resulted in an important decrease in survival for ds*ApPAH*-treated aphids (84.4% and 25.5% survival rate at day 14 for 0.75 μg/μL and 5.0 μg/μl dsRNA, respectively). The control dsEGFP group also showed some mortality (95.6% and 73.3% survival rate at day 14 for 0.75 μg/μL and 5.0 μg/μl dsRNA, respectively). The mortality was, at all dsRNA concentrations, always significantly greater in the ds*ApPAH*-injected aphids compared to the dsEGFP control group, demonstrating a specific effect of the ds*ApPAH* treatment on aphid survival (Fig. 5c).

ds*ApPAH* treatment did not induce any visible morphological or obvious behavioral phenotype in the parthenogenetic mothers, but many morphological defects were observed in their progeny. To investigate the possible effect of ds*ApPAH* treatment on aphid fecundity and embryonic development, a complete RNAi study was designed using an optimal ds*ApPAH* concentration (1.8 μg/μl) that affected aphid survival whilst still allowing for sufficient numbers of viable mothers. This, in turn, enabled us to study the consequences of maternal dsRNAi treatment on newborn nymphs.

### *ApPAH* gene expression inhibition following RNAi treatment

Since dsRNA silencing in aphids induces non-systemic and variable individual responses^39^, the *ApPAH* expression level following RNAi-mediated treatment was analyzed, using qRT-PCR, in four different tissues isolated from individual aphids injected with the selected concentration of 1.8 μg/μl (Fig. 6). The qRT-PCR analysis revealed that *ApPAH* knockdown specifically targeted two aphid body compartments, the specialized endosymbiont-bearing cells (bacteriocytes) and the embryonic chains. Specifically, *ApPAH* expression was significantly reduced 24 h and 72 h after injection in bacteriocytes (72% and 70% reduction, respectively; Student’s t-test, P < 0.05) and in embryonic chains (90% and 78% reduction, respectively; Student’s t-test, P < 0.001), compared to the dsEGFP negative control. No significant impact of RNAi-mediated treatment on the *ApPAH* transcript level was observed in the gut or the carcass (including the head).

### Effects of *ApPAH* RNAi on aphid fecundity

The *ApPAH* knockdown considerably affected aphid fecundity. The total number of nymphs produced was significantly reduced compared to the controls (Fig. 7a). Over the 14 day period following injections, 10 aphids in groups treated with RNase-free water, dsEGFP and ds*ApPAH* laid, on average, 595.7, 510.0 (−14.4%) and 412.0 (−30.8%) nymphs, respectively. Interestingly, in line with the observed *ApPAH* inhibition in the embryonic chains, fecundity analysis also showed that ds*ApPAH* treatment resulted in a significant increase in defective progeny (viable and/or non-viable) (Fig. 7b; Tukey’s HSD test, P < 0.001). In comparison, 11.8% of the newborn nymphs of ds*ApPAH*-injected aphids showed defects at 14 days after injection, compared with only 0.5% or 0.9% for the RNase-free water or dsEGFP control groups, respectively. In the ds*ApPAH*-treated group, defective nymphs were observed as early as day 6 after injection while in control groups this was observed much later, i.e. from day 13 (Fig. 7c).

### Effects of *ApPAH* RNAi on embryonic development and tyrosine biosynthesis

Morphological analysis of defective nymphs showed impaired embryonic development following *ApPAH* knockdown (Fig. 8a). Two phenotypic classes were distinguished, based on the severity of the morphological deformations.

Class I nymphs (15% of defective nymphs) exhibited moderate developmental defects (Fig. 8a, B1-B2). In this phenotype, the labium and the antennae were most frequently formed but the legs were affected, showing vestigial or altered morphologies. We consistently noted the presence of correctly formed eyes. Finally, we observed a body compaction resulting in a significantly reduced size of this nymph class, compared to the wild type (Fig. 8b; Student’s t-test, P < 0.001).

Class II nymphs, the strongest phenotypic class, was made up of 85% of the defective nymphs laid after ds*ApPAH* treatments (Fig. 8a, C1-C2). Contrary to class I, nymph appendages, including antennae, labial segments or thoracic legs, were considerably reduced or completely absent such that these nymphs resembled intermediate or late stage embryos, still alive but blocked in their embryonic development. Eye development was variable, ranging from recognizable and correctly formed eyes (Fig. 8a, C1) to undeveloped organs (Fig. 8a, C2). These nymphs showed a more severe reduction in body length than the phenotypic class I (Fig. 8b).

Single nymph HPLC analyses confirmed that the impairment of embryonic development, resulting from *ApPAH* knockdown, was directly linked to the disruption of tyrosine biosynthesis (Fig. 8c). Compared to the controls, class I and class II defective nymphs showed a significantly decreased relative concentration of tyrosine (1.9- and 2.0-fold, respectively) as well as a consistent increase in phenylalanine (1.4- and 2.1-fold, respectively), the upstream precursor of tyrosine, with a gradient effect correlated with the severity of the phenotypes.

## Discussion

Compared to mammals and other species, insects have a highly evolved and more diversified system of tyrosine metabolic enzymes^40^. This dominant class of terrestrial metazoans by diversity of species, habitats and lifestyles, relies on tyrosine metabolism for a broad range of physiological processes, including insect neuromodulation, pigmentation, cuticle sclerotization, eggshell tanning and immune responses^41^,^42^,^43^,^44^. Interestingly, in many symbiotic insects that depend on their mutualistic prokaryotic partners for the production of essential nutrients, such as vitamins or amino acids, convergent evolution processes lead to strong host/symbiont collaboration for tyrosine biosynthesis: symbionts supply the insect with tyrosine metabolic precursors (prephenate, phenylpyruvate or phenylalanine) which the host then transforms into tyrosine^45^,^46^.

**Figure 1:**
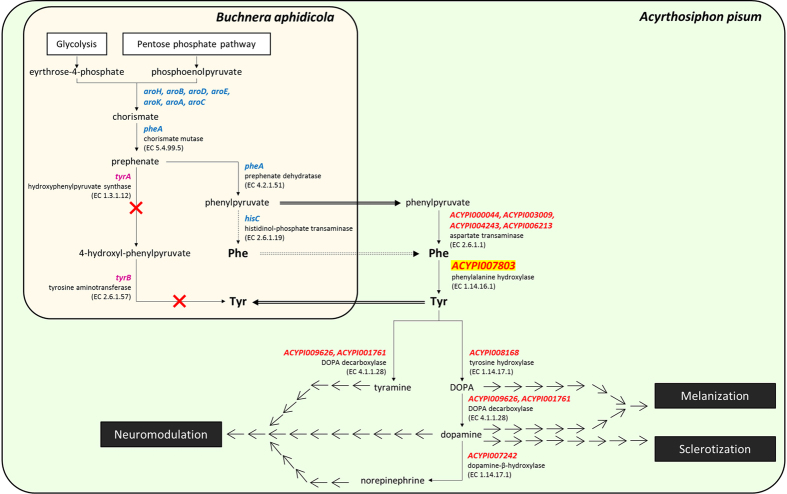
Overview of tyrosine metabolism in *A. pisum*/*B. aphidicola* symbiosis. Schematic representation of the tyrosine integrated pathway between the pea aphid (light green rectangle) and its primary endosymbiont (light beige rectangle). Enzymes are indicated with their Enzyme Commission (EC) numbers at the corresponding reactions: red, pea aphid enzyme-coding genes; blue, *Buchnera* enzyme-coding genes; purple, *Buchnera* missing genes. Single line and double line arrows represent metabolic reactions and transport steps, respectively. Solid arrows indicate annotated events whereas dotted arrows refer to possible events. Abbreviations: Phe, phenylalanine; Tyr, tyrosine. Figure adapted from Rabatel *et al*.^34^.

Tyrosine metabolism in the *A. pisum*/*B. aphidicola* symbiotic system is a striking example and can be considered as a model for mutual metabolic interdependence, consisting of an integrated network of genes encoded by both partner genomes^36^,^47^,^48^. *B. aphidicola*, whose central metabolism is based on glucose and mannitol utilization via the glycolysis and the pentose phosphate pathways, is genetically able to synthesize the carbon skeleton of tyrosine with the production of the precursor phenylpyruvate. However, it lacks the bacterial genes required for the terminal tyrosine-biosynthesis reactions (*tyrA* and *tyrB*). It is the pea aphid genome that fills these gaps in the tyrosine biosynthetic pathway, encoding putative genes for aspartate transaminase (*ACYPI000044*, *ACYPI003009*, *ACYPI004243* and *ACYPI006213*; EC 2.6.1.1) and phenylalanine hydroxylase (*ACYPI007803*; EC 1.14.16.1) (Fig. 1).

In the present study, we focused on the characterization of *ApPAH* (*ACYPI007803*), the only gene of the pea aphid genome putatively encoding for phenylalanine hydroxylase enzymatic activity. Contrary to the other enzymes of the pathway, such as aspartate transaminase, which is also potentially involved in alanine/aspartate/glutamate, cysteine/methionine or arginine/proline metabolism, phenylalanine hydroxylase is restricted to the tyrosine pathway. Hence, targeting the *ApPAH* gene is expected to specifically interfere with tyrosine production, providing an opportunity to identify new tyrosine-associated functions in insect development.

Despite a high variability of animal *PAH* exon-intron structures, ApPAH shares high amino acid sequence similarity with the hitherto identified metazoan PAH proteins, higher than 59% and 64% with mammals and insects, respectively. Sequence alignment and phylogenetic analysis confirmed that ApPAH is closely related to insect PAH, and clusters with orthologs of hemimetabolous insects suggesting evolutionary relatedness and, possibly, similar physiological functions. A detailed analysis of the ApPAH protein sequence further demonstrated the presence of both an ACT regulatory domain and a catalytic domain, including binding sites for amino acids, iron, the substrate and the BH_4_ cofactor. These two interacting domains^2^,^49^ are well conserved among all characterized PAH proteins, illustrating the key role they play in enzymatic activity. Structure-disturbing mutations in the ACT regulatory domain have been demonstrated to disrupt the functionality of the human PAH enzyme^50^.

*ApPAH* is expressed throughout the pea aphid life cycle, reaching the highest expression levels in late embryonic and first nymphal stages. Different expression profiles have been previously reported for holometabolous insects with, notably, an increase in *PAH* expression throughout embryonic and larval development in the fruit fly *D. melanogaster*^14^ and a maximum expression level in the *A. aegypti* adult female mosquitoes^31^. Hence, the developmental expression pattern of *ApPAH* is indicative of a key role for this gene in pea aphid parthenogenetic embryonic development. This strengthens the hypothesis of diverse PAH-mediated processes in metamorphic and reproductive life strategies other than those previously characterized in holometabolous species with oviparous reproduction. Using our maternal RNAi-based approaches, we were able to demonstrate an essential role of *ApPAH* during parthenogenetic development. The *ApPAH* silencing led to a significantly reduced fecundity in pea aphids. Further examination of the offspring demonstrated that *ApPAH* knockdown interferes with parthenogenetic embryo development, affecting eyes and appendages (antennae, labial segments and legs) and provoking a body compaction that rendered the limit between thoracic and abdominal segments almost undistinguishable. The observation of nymphs blocked at a more or less advanced developmental stage, following RNAi treatments, can be attributed to differences in both (i) the levels of gene knockdown between embryos (i.e. the strongest phenotypes would result from the highest inactivation levels) and (ii) the developmental stage at which embryos are targeted by RNAi (i.e. in the same embryonic chain, early embryos would suffer the most significant developmental defects whereas late embryos, having almost completed their development, would be less severely affected).

This study is the first report demonstrating a direct effect of *PAH* inactivation on the embryonic development of insects. Previous studies in holometabolous insects have shown that *PAH* knockdown predominantly disrupted melanin-associated processes in egg, larval and adult stages. For example, in the lepidopteran model *B. mori*, *PAH* knockdown experiments, following injections of dsRNA into silkworm eggs, resulted in a failure of body coloration in neonatal larvae^18^. Moreover, whereas *PAH* transcripts have been shown to increase in the mosquito hemolymph following immune-stimulation^30^,^31^, *PAH* knockdown in *A. aegypti*, *A. gambiae* and *A. subalbutus* provoked a significant reduction in the melanization response, a unique feature of insect immunity, against filarial worms and protozoan parasites^17^,^32^. Finally, *PAH* inactivation in mosquitoes and silkworms led to a reduced oviposition rate, chorion maturation and egg hatchability^18^,^32^. As far as we know, no studies have yet been published on PAH functions in hemimetabolous insects.

The significant reduction in the tyrosine level, observed in nymphs with developmental defects (both class I and class II), points to a direct link between these phenotypes and the disruption of this amino acid biosynthesis mediated by ApPAH activity. A deficiency of tyrosine is expected to alter the production of tyrosine metabolic derivatives, such as melanin or catecholamines, essential for exoskeleton formation, eye and body pigmentation and neurotransmission^41^,^42^,^43^,^44^. Since insect exoskeleton synthesis is a precondition for the formation of the locomotor apparatus and other appendages, we propose that the vestigial and deformed appendages observed in ds*ApPAH*-defective nymphs are directly due to an alteration in exoskeleton formation caused by a lack of tyrosine, and/or its derivatives. Furthermore, since *B. aphidicola*, the pea aphid primary endosymbiont, relies on its host for tyrosine supply^34^,^36^, we speculate that *ApPAH* knockdown in developing embryos might also disturb the nutritional interactions between the two symbiotic partners, possibly resulting in serious developmental side-effects. Finally, the accumulation of phenylalanine we observed in ds*ApPAH* defective nymphs is consistent with *PAH* knockdown, as this amino acid is the direct precursor of tyrosine. Such high levels of phenylalanine could be toxic for pea aphid embryonic development, as described for phenylketonuria (PKU) in mammalian models^20^.

In summary, the present work has characterized, for the first time, the role of phenylalanine hydroxylase in a hemimetabolous model insect, the pea aphid *Acyrthosiphon pisum*. While PAH involvement in post-embryonic development and adult physiological processes has been previously demonstrated, our results allow extending the knowledge of PAH-associated functions to insect embryonic development.

## Methods

### Sequence analysis and phylogeny

The *ApPAH* gene sequence was obtained from the *A. pisum* genomic database AphidBase^38^ (v2.1) (https://www.aphidbase.com) and located from nucleotides 185,331 to 193,162 on the scaffold GL350506. The 3′ UTR region of the predicted *ApPAH* transcript (*ACYPI007803-RA*; 3,509 nt) was re-annotated based on the identification of a unique canonical polyadenylation signal (AATAAA), spanning nucleotides 186,570 to 186,575, and the downstream polyadenylation site (CA) at the genomic positions 186,536 and 186,537, shortening the length of the transcript originally predicted to 2,304 nt. The 37 RNA-seq data libraries available from AphidBase supported this re-annotation. The domains of the deduced protein sequence were identified using the NCBI Conserved Domain Search tool (http://www.ncbi.nlm.nih.gov/Structure/cdd/cdd.shtml)^51^. Orthologous PAH proteins were retrieved using BLASTP against the NCBI non-redundant protein database (see Supplementary Table S1 for a complete list of orthologous sequences used for the phylogenetic analysis). ApPAH was subjected to multiple sequence alignments using the MUSCLE program^52^. Based on the MUSCLE-derived alignment, a site was considered as being informative, and then selected for the phylogenetic reconstruction, when at most two species were exhibiting a gap in the multiple sequence alignment. This site selection was performed using the SeaView software^53^. Subsequently, a phylogenetic tree was constructed, using the PhyML method^54^ (LG model with 4 rate classes, 439 informative sites analyzed), and the reliability of each branch was evaluated using the bootstrap method, with 1000 replications. Poorly supported branches (<50%) were collapsed using TreeCollapseCL 4 (http://emmahodcroft.com/TreeCollapseCL.html).

### Aphid rearing

A long-established parthenogenetic clone (LL01) of *A. pisum* Harris was maintained on young broad bean plants (*Vicia faba* L. cv. Aguadulce) at 21 °C, with a photoperiod of 16 h light/8 h dark. To obtain a source of synchronized aphids, winged adults were left on seedlings, allowing them to produce nymphs, and were removed after 24 h. Synchronized N1 nymphal instars were left on plants and sampled at different stages, depending on the experiments.

### Sampling for developmental analysis of *ApPAH* mRNA expression

Aphids were collected, as in Simonet *et al*.^55^, at the following life stages: early embryos EE (≤0.4 mm), intermediate embryos IE (0.4 to 0.8 mm) and late embryos LE (>0.8 mm), corresponding, respectively, to embryonic stages ≤15, 16–18 and 19–20 as described by Miura *et al*.^37^; nymphs N1 (first instars; 1 day old), N2 (second instars; 2 days old), N3 (third instars; 5 days old), and N4 (fourth instars; 7 days old); and adults at three distinct time points: A8 (8 days old), A15 (15 days old) during the reproductive period, and A23 (23 days old) during the aphid aging period. All of the collected nymphs and adults were randomly selected from the synchronized source population. Embryos were dissected from 13-day-old adults. For each replicate, all the EE embryos were collected from three aphids; three IE or LE embryos were taken from three different aphids; and three aphids were collected for each of the N1-A23 life stages. These were then placed in RNA*later*^®^ solution (ThermoFisher Scientific, Waltham, MA, USA) and stored at −80 °C until qRT-PCR analyses were performed. For each life stage, three independent replicates were processed.

### dsRNA synthesis

**Figure 2:**
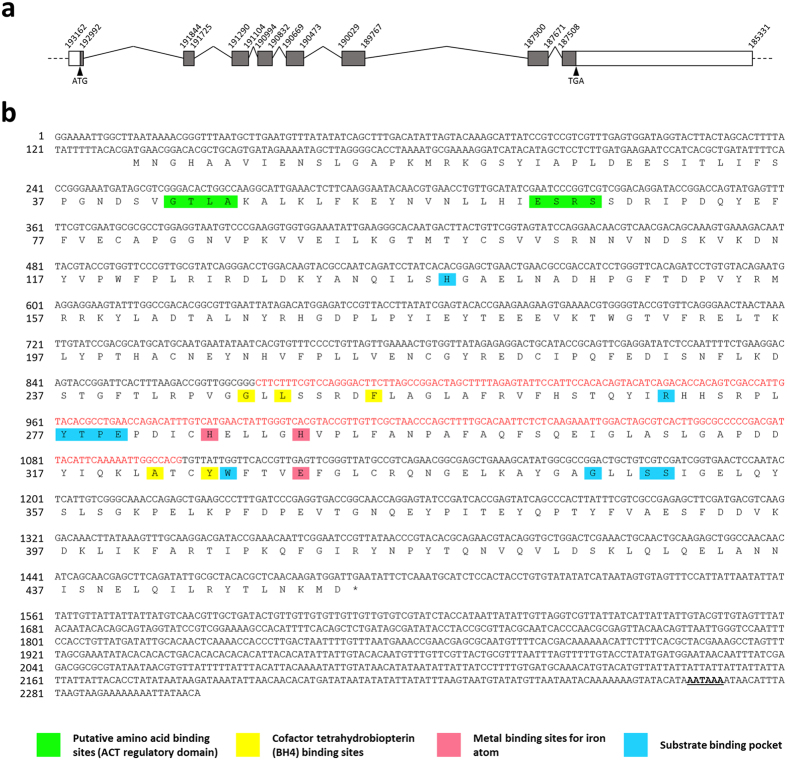
Characterization of the *ApPAH* gene. (**a**) Schematic overview of *ApPAH* gene structure. Exons, 5′ and 3′ untranslated region (UTR), and introns are represented with grey boxes, white boxes, and solid lines, respectively. Arrowheads indicate the translation start site (ATG) and stop codon (TGA). Genomic coordinates refer to the positions on the scaffold GL350506 available from AphidBase^38^. (**b**) Nucleotide and amino acid sequences of the *ApPAH* gene. *ApPAH* comprises a 1,359 bp CDS encoding 452 amino acid residues, indicated as single-letters above the nucleotide sequence. Conserved binding domains are highlighted in different colors. The stop codon is marked with an asterisk. Underlined bold letters and red letters indicate the polyadenylation signal (AATAAA) and the sequence targeted by the ds*ApPAH*, respectively.

**Figure 3:**
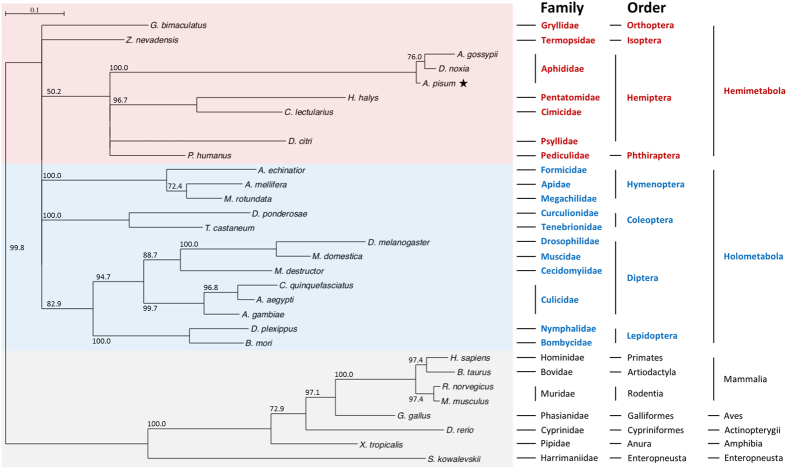
Unrooted phylogenetic tree of PAH proteins. Maximum-likelihood phylogenetic tree reconstruction was performed using the SeaView software^53^, according to a PhyML method^54^ with an LG 4-rate class model. Branch-support values were calculated by the bootstrap method with 1000 replications, and displayed at each inner node. Branches with bootstrap values below 50% were collapsed. Insect orders were positioned according to the insect phylogeny determined by Misof *et al*.^58^. The sequences (with GenPept accession numbers) used for the phylogenetic analysis are listed in Supplementary Table S1.

**Figure 4:**
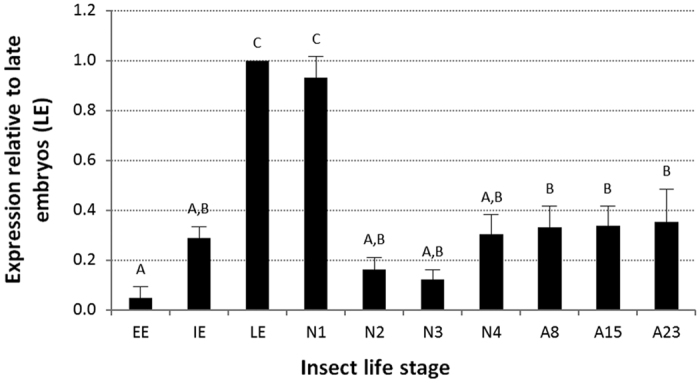
mRNA expression profile of *ApPAH* during aphid development. qRT-PCR analysis of *ApPAH* expression at different life stages, relative to the expression in late embryos (corresponding to the maximum expression value). The *rpl7* and *actin* genes were used for data normalization. Results are reported as means ± SD; n = 3 independent biological replicates per stage (each biological replicate was composed of all of the EE embryos from three aphids, three IE or LE embryos, or three aphids for the N1-A23 life stages). Data were analyzed by one-way ANOVA followed by a *post hoc* multiple comparisons test (Tukey’s HSD test). Life stages labeled with different letters are significantly different (P < 0.05). Abbreviations: EE, early embryos; IE, intermediate embryos; LE, late embryos; N1 to N4, nymphal stages from 1 to 4; A8-A23, adult time points from day 9 to day 23.

**Figure 5:**
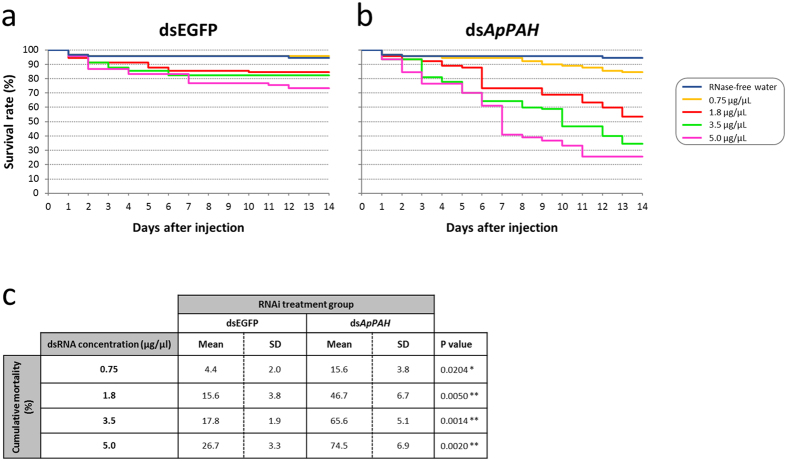
Impact of ds*ApPAH* on aphid survival. (**a,b**) Survival curves of aphids injected with dsEGFP (**a**) or ds*ApPAH* (**b**), at different dsRNA concentrations. Results are reported as means, n = 3 independent biological replicates (30 aphids per biological replicate). (**c**) Cumulative mortality of RNAi treated-aphids. Results are reported as means ± SD, n = 3 independent biological replicates (30 aphids per biological replicate). For each dsRNA concentration, significant differences between dsEGFP and ds*ApPAH*-treated groups were analyzed by a Student’s t-test and are indicated with asterisks (*P < 0.05; **P < 0.01).

**Figure 6:**
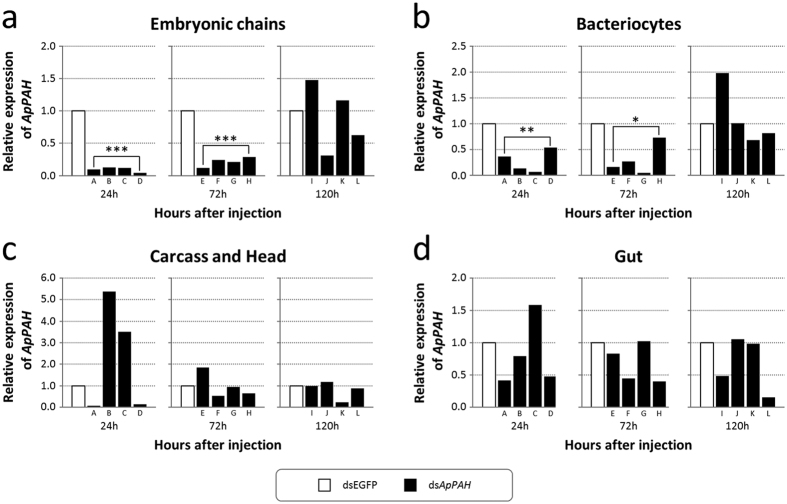
Knockdown analysis of *ApPAH* following dsRNA injection. (**a–d**) qRT-PCR determination of *ApPAH* expression levels in the embryonic chains (**a**), bacteriocytes (**b**), carcass and head (**c**), and gut (**d**) of ds*ApPAH*-treated aphids, relative to expression levels of the dsEGFP controls. Each body compartment was analyzed from four individual aphids at 24 h (labeled A, B, C and D), 72 h (E, F, G and H), and 120 h (I, J, K and L) post-ds*ApPAH* injection. The *rpl7* and *actin* genes were used for data normalization. Data were analyzed with a unilateral Student’s t-test for down-regulations and significant results are indicated with asterisks (*P < 0.05; **P < 0.01; ***P < 0.001).

**Figure 7:**
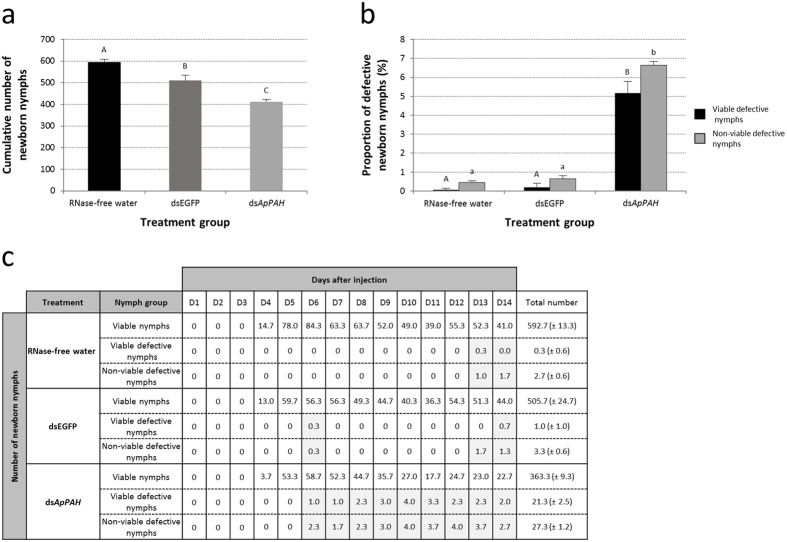
Impact of ds*ApPAH* on aphid fecundity. (**a**) Cumulative number of progeny (at day 14) laid by the different treatment groups: aphids injected with RNase-free water (black), dsEGFP (grey) or ds*ApPAH* (light grey), respectively. For each treatment group, results are reported as means (±SD) of three independent biological replicates. Each biological replicate was composed of one group of 10 aphids. Data were analyzed by one-way ANOVA followed by a *post hoc* multiple comparisons test (Tukey’s HSD test). Treatment groups labeled with different letters are significantly different (P < 0.05). (**b**) Proportion of viable defective (black) and non-viable defective (light grey) nymphs laid by the different treatment groups: aphids injected with RNase-free water, dsEGFP or ds*ApPAH*, respectively. For each treatment group, results are reported as means (±SD) of three independent biological replicates. Each biological replicate was composed of one group of 10 aphids. Data were analyzed by one-way ANOVA followed by a *post hoc* multiple comparisons test (Tukey’s HSD test). Treatment groups labeled with different letters are significantly different (P < 0.05). (**c**) Variation in the number of newborn nymphs in relation to the different RNAi treatments: RNase-free water, dsEGFP or ds*ApPAH*. Newborn nymphs were classified into three groups: viable, viable defective and non-viable defective nymphs. Results are reported as means (day by day report), or as means ± SD (cumulative number of newborn nymphs at the end of the follow-up period) of three independent biological replicates. Days with defective nymph production are highlighted in grey.

**Figure 8:**
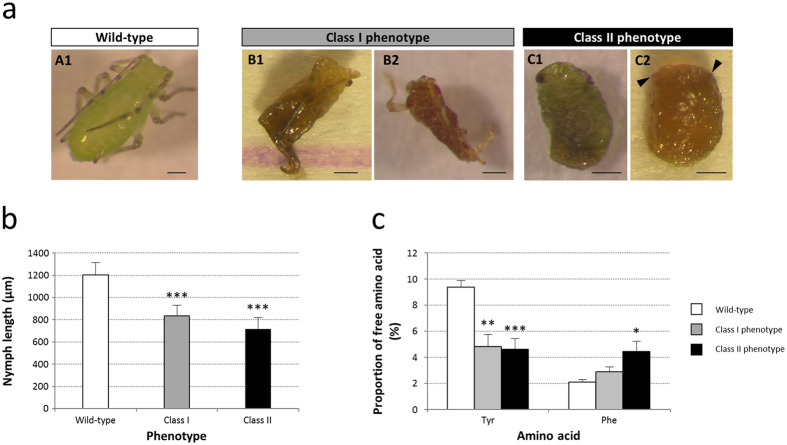
Nymph phenotypes induced by parental *ApPAH* RNAi. (**a**) Morphological alteration of defective nymphs laid by ds*ApPAH*-injected aphids. Two nymph classes were distinguished based on the apparent severity of their morphological phenotypes, compared to the wild type (A1). B1-B2: Class I “moderate” phenotype. Note the abnormal body shape and the deformed or vestigial thoracic legs. C1-C2: Class II “severe” phenotype. Note the defective development of nymph appendages, including antennae, labium and thoracic legs, and eye abnormalities. Arrowheads indicate missing or underdeveloped eye structures. Scale bars = 200 μm. (**b**) Variation in nymph length in relation to phenotypes. Results are reported as means ± SD, n > 10 nymphs per phenotype. Data were analyzed by one-way ANOVA followed by a *post hoc* multiple comparisons test (Tukey’s HSD test). Significant differences, compared to wild type, are indicated with asterisks (***P < 0.001). (**c**) Single nymph HPLC analyses of free tyrosine and phenylalanine. Results are expressed as a percentage of the total amount of amino acids in the different phenotypes and reported as means ± SD, n = 8 nymphs per phenotype. Data were analyzed by one-way ANOVA followed by a *post hoc* multiple comparisons test (Tukey’s HSD test). Significant differences, compared to the wild type, are indicated with asterisks (*P < 0.05; **P < 0.01; ***P < 0.001).

The *ApPAH* and EGFP target sequences (i.e. regions that showed no similarities with other transcripts or low-complexity regions in the pea aphid genome) were selected using the E-RNAi webtool (http://www.dkfz.de/signaling/e-rnai3//)^56^. We specifically targeted exon 6 of the *ApPAH* gene, which contains most amino acids involved in cofactor, metal and substrate binding (Fig. 2). dsRNA templates were obtained by PCR with gene-specific primers containing a T7 promotor sequence (see Supplementary Table S2), as previously described^39^. dsRNA was synthesized using the MEGAscript T7 kit (ThermoFischer Scientific, Waltham, MA, USA), according to the manufacturer’s instructions. The dsRNA products were then purified using the RNeasy mini kit (Qiagen, Hilden, Germany), quantified with a Nanodrop ND-1000 spectrophotometer (Nanodrop technologies, Wilmington, DE, USA), and their quality was verified using an Agilent 2100 Bioanalyzer (Agilent Technologies, Santa Clara, CA, USA).

### dsRNA injection

Synchronized 3^rd^ nymphal instars (N3) were injected with dsRNA, following the procedure described by Sapountzis *et al*.^39^. Briefly, in order to minimize the mortality associated with microinjections, 46 nl of dsRNA was injected between the 2^nd^ and the 3^rd^ abdominal segment using an automatic injector apparatus Nanoject II (Drummond Scientific, Broomall, PA, USA) with 1.0 mm O.D. × 0.78 mm I.D. capillaries. Aphids were immobilized with a home-made vacuum-operated insect-holder for accurate positioning of the aphids for intra-abdominal injections. dsRNA was administered at 1.8 μg/μl, the highest concentration able to induce effects specific to the target gene without inducing high mortality rates.

### Analysis of phenotypes following dsRNA treatment

After the injections, aphids were divided into three treatment groups (injected with RNase-free water, dsEGFP or ds*ApPAH)* and reared under standard conditions for a visual monitoring of the phenotypes and subsequent analyses of the life history traits (see Supplementary Fig. S2).

Over a 14-day post-injection period, for each treatment group, 30 aphids were checked daily for survival and 10 aphids were followed individually for fecundity. Three independent experiments were performed. The number of newborn nymphs (viable, viable defective or non-viable defective) was recorded under a light microscope. Microscopy images and length measurements of newborn nymphs were performed with a Leica MZ FL III stereomicroscope (Leica, Wetzlar, Germany) equipped with an Olympus XC50 color camera linked to the CellF software. Among defective nymphs, two phenotypic classes (class I and class II), based on the severity of the morphological deformations, were distinguished and analyzed by HPLC for their amino acid content. For each phenotypic class, eight independent replicates were processed. The effects of dsRNA on *ApPAH* expression were analyzed by qRT-PCR. Four aphids were collected from each treated-group at 24, 72 and 120 h after microinjections, respectively. Four body compartments were carefully dissected and isolated from each individual aphid in ice-cold buffer A (0.025 M KCl, 0.01 M MgCl_2_, 0.25 M Sucrose, and 0.035 M Tris-HCl, pH 7.5): the bacteriocytes, the gut, the embryonic chains (produced by parthenogenesis in the asexual viviparous aphids used in this study) and the rest of the body, including the remaining carcass and the head. All dissections were performed under 25X-40X magnification with a MDG-17 stereomicroscope (Leica, Wild Heerbrugg AG, Switzerland). The tissues were placed in RNA*later*^®^ solution and stored at −80 °C until qRT-PCR analyses were performed.

### Real-time quantitative RT-PCR

Total RNA was extracted from whole aphids or dissected tissues using the RNeasy mini kit. The RNA was treated with DNase I (Promega, Madison, WI, USA) and first strand cDNA was synthesized using the Sensiscript RT Kit (Qiagen, Hilden, Germany) with oligo(dT) primers (ThermoFischer Scientific, Waltham, MA, USA). Real-time RT-PCR reactions were performed on a LightCycler^®^ 480 instrument (Roche, Basel, Switzerland) using 1:2.5 diluted cDNAs and SYBR Green PCR Master mix, according to the manufacturer’s instructions. *ApPAH* mRNA levels were quantified relative to constitutively expressed *rpl7* (*ACYPI010200*) and *actin* (*ACYPI000064*). These two genes were retained by the BestKeeper software tool^57^ as the best normalization genes compared to other candidates: *cyclophilin* (*ACYPI003541)*, *gadph* (*ACYPI008372*) and *rpl32* (*ACYPI000074*). Primers used in this study are listed in Supplementary Table S2. All measurements were performed in triplicate and relative *ApPAH* expression levels were calculated as previously described^39^.

### Single nymph HPLC analysis

Free amino acid HPLC analysis was carried out on individual nymphs by adapting the procedure specifically developed for pea aphid embryos and nymphs^34^. Amino acid analysis was performed by HPLC (Agilent 1100; Agilent Technologies, Santa Clara, CA, USA) with a guard cartridge and a reverse phase C18 column (Zorbax Eclipse-AAA 3.5 μm, 150 × 4.6 mm, Agilent Technologies). The software used was the ChemStation for LC 3D Systems (Agilent Technologies).

### Statistics

All statistical analyses were carried out using the R software v3.1.1, with values of P < 0.05 considered as being significant. Data normality and homoscedasticity assumptions were checked with the Shapiro-Wilk and Bartlett tests, respectively. The differences in *ApPAH* expression levels among pea aphid life stages, cumulative offspring production and the proportion of defective nymphs among the RNAi-treated groups, together with nymph length and the relative amino acid concentration of the RNAi-associated nymph phenotypes, were all analyzed using one-way analysis of variance (ANOVA), followed by *post hoc* multiple comparisons using Tukey’s HSD test. Any significant variation in aphid mortality between the RNAi-treated groups, and in RNAi knockdown in the pea aphid body compartments, was determined with the Student’s t-test.

## Additional Information

**How to cite this article**: Simonet, P. *et al*. Disruption of phenylalanine hydroxylase reduces adult lifespan and fecundity, and impairs embryonic development in parthenogenetic pea aphids. *Sci. Rep*. **6**, 34321; doi: 10.1038/srep34321 (2016).

## Supplementary Material

Supplementary Information
