# Supplementary material for: Disruption of phenylalanine hydroxylase reduces adult lifespan and fecundity, and impairs embryonic development in parthenogenetic pea aphids
Source: Sci Rep. 2016 Oct 3;6:34321. doi: 10.1038/srep34321 (PMC5046115; doi:10.1038/srep34321)
Supplement: Supplementary Information [file srep34321-s1.pdf]

Disruption of phenylalanine hydroxylase reduces adult lifespan and fecundity, and impairs embryonic development in parthenogenetic pea aphids

Pierre Simonet[1]*, Karen Gaget[1], Nicolas Parisot[1], Gabrielle Duport[1], Marjolaine Rey[1,§], Gérard Febvay[1], Hubert Charles[1], Patrick Callaerts[2,3], Stefano Colella[1] and Federica Calevro[1]*

[1] Univ Lyon, INSA-Lyon, INRA, BF2I, UMR0203, F-69621, Villeurbanne, France. [2] KU Leuven, University of Leuven, Department of Human Genetics, Laboratory of Behavioral and Developmental Genetics, B-3000, Leuven, Belgium. [3] VIB Center for the Biology of Disease, B-3000, Leuven, Belgium.

*Corresponding authors: pierre.simonet@insa-lyon.fr and federica.calevro@insa-lyon.fr

[§] Present address: Univ Lyon, CNRS, INRA, Ecologie Microbienne, UMR5557, F-69622, Villeurbanne, France.

Tel.: +33 472 43 79 88

Fax: +33 472 43 85 34

Supplementary Information

Supplementary Figure S1. Microphotographs representing parthenogenetic pea aphids and embryonic chains. (a) Adult and nymphal females on *Vicia faba* plants. (b) Ovaries dissected from a parthenogenetic viviparous female. Each female contains two ovaries composed of 6-7 embryonic chains in which tens of genetically identical embryos develop sequentially. Abbreviations: Ad, adults; EC, embryonic chain; LE, late embryos; N, nymphs.

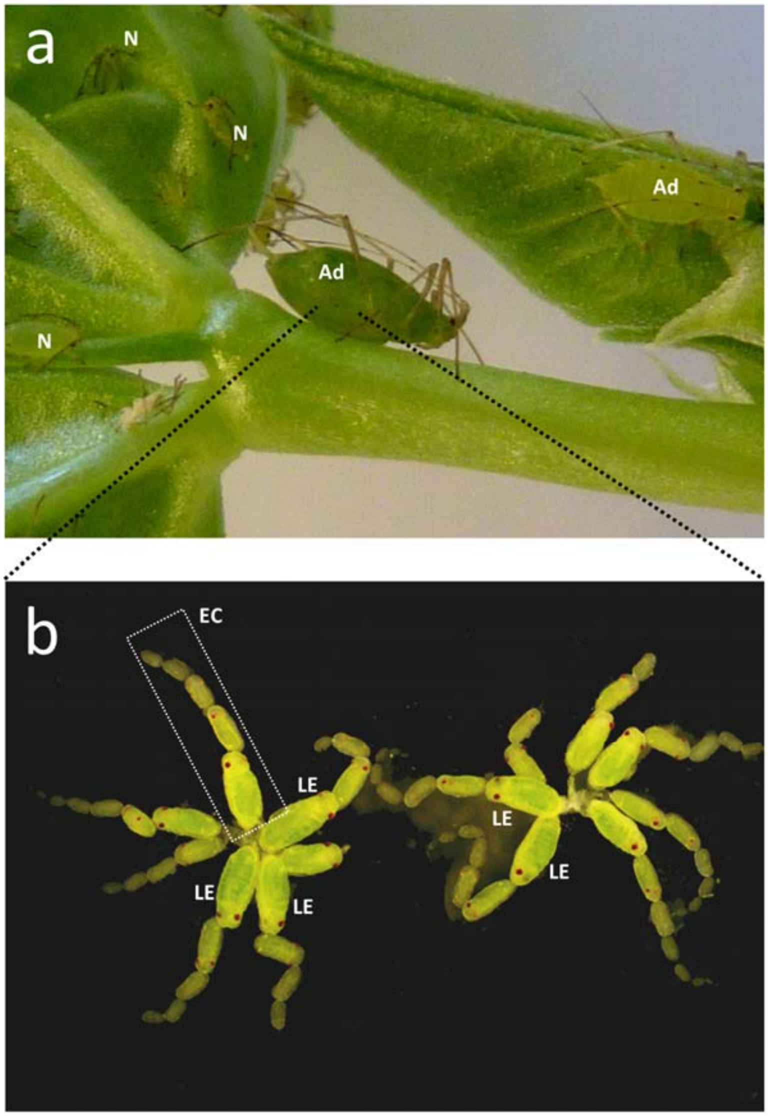

Supplementary Figure S2. Schematic representation of RNAi-associated experiments. This diagram illustrates the sampling strategy for each experiment following dsRNA treatment by microinjection in N3 pea aphids.

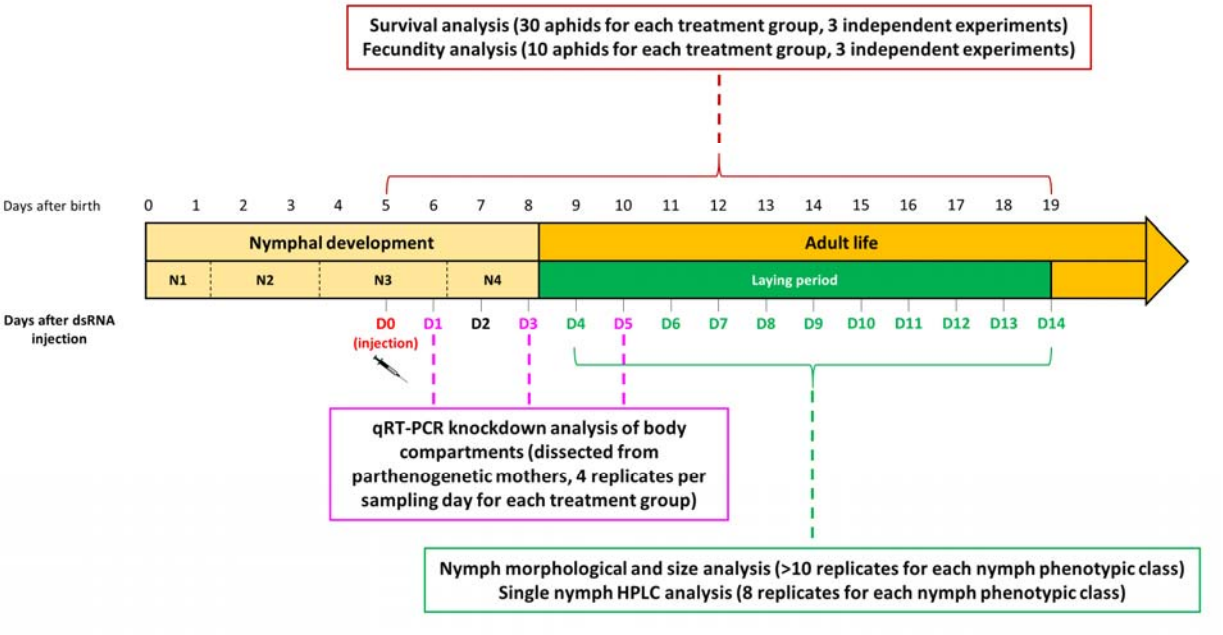

Supplementary Table S1. PAH sequences used for phylogenetic analysis. The sequences are ordered according to phylogenetic proximity to pea aphid PAH (as in Fig. 3). Abbreviation: NA, not available.

GenPept accession number	Species	Exon number	Percent identity[1] (%)
BAJ83477.1	*Gryllus bimaculatus*	NA	67
KDR21968.1	*Zootermopsis nevadensis*	8	70
AML23854.1	*Aphis gossypii*	NA	94
XP_015370421.1	*Diuraphis noxia*	8	97
XP_014289709.1	*Halyomorpha halys*	10	68
XP_014252769.1	*Cimex lectularius*	8	69
XP_008487883.1	*Diaphorina citri*	10	74
XP_002429284.1	*Pediculus humanus corporis*	7	73
EGI62058.1	*Acromyrmex echinatior*	8	67
XP_623300.1	*Apis mellifera*	9	71
XP_003705333.1	*Megachile rotundata*	11	69
ERL87023.1	*Dendroctonus ponderosae*	10	65
XP_967025.1	*Tribolium castaneum*	6	65
NP_523963.2	*Drosophila melanogaster*	6	67
XP_005184623.1	*Musca domestica*	6	67
AHB50504.1	*Mayetiola destructor*	NA	69
XP_001843416.1	*Culex quinquefasciatus*	3	67
XP_011493674.1	*Aedes aegypti*	4	66
XP_001688715.1	*Anopheles gambiae*	5	64
EHJ64587.1	*Danaus plexippus*	6	67
XP_004924935.1	*Bombyx mori*	7	67
NP_000268.1	*Homo sapiens*	13	62
NP_001039523.1	*Bos taurus*	13	62
NP_036751.2	*Rattus norvegicus*	14	59
NP_032803.2	*Mus musculus*	13	60
NP_001001298.1	*Gallus gallus*	13	66
NP_956845.1	*Danio rerio*	13	62
NP_001027484.1	*Xenopus tropicalis*	13	60
NP_001161629.1	*Saccoglossus kowalevskii*	12	62

[1]Percent identity = percentage of amino-acid sequence identical to ApPAH protein sequence.

Supplementary Table S2. Primers used for qRT-PCR analysis and RNAi experiments.

Gene	ACYPI mRNA ID	Primer	Sequence	Amplicon length (bp)
For qRT-PCR				
actin	*ACYPI000064-RA*	Forward (F)	5'- -3' AAGTTATCACAATCGGAAATG	196
		Reverse (R)	5'- -3' GGCAATACCAGGGTACAT	
cyclophilin	*ACYPI003541-RA*	Forward (F)	5'- -3' AGACATCGTGGTTGGA	186
		Reverse (R)	5'- -3' TTTAATTGATACGTTTGACAT	
gapdph	*ACYPI008372-RA*	Forward (F)	5'- -3' TGGCTTCAACTAACTACCAAC	128
		Reverse (R)	5'- -3' TGATTTTTACTTTGCGGACT	
PAH	*ACYPI007803-RA*	Forward (F)	5'- -3' TTCACGACAAAAAACATTCTTTC	124
		Reverse (R)	5'- -3' AAATTAAACGCAGTAACGAAC	
rpl7	*ACYPI010200-RA*	Forward (F)	5'- -3' TCAAGGGACAACGCATTC	142
		Reverse (R)	5'- -3' CAAAGGAAGTTCATCGCATAC	
rpl32	*ACYPI000074-RA*	Forward (F)	5'- -3' AGTATCGCCCAACAATTATCA	130
		Reverse (R)	5'- -3' CTTGAATCGTCTTCGGACT	
For dsRNA synthesis				
EGFP	-	Forward (F)	5'- -3' taatacgactcactatagggCCCTCGTGACCACCCTGACC	-
		Reverse (R)	5'- -3' taatacgactcactatagggTTCTCGTTGGGGTCTTTGCT	
PAH	*ACYPI007803-RA*	Forward (F)	5'- -3' taatacgactcactatagggCTTCTTTCGTCCAGGGACTTC	268
		Reverse (R)	5'- -3' taatacgactcactatagggCGTGGCCAATTTTTGAATGT	
